# Supplementary material for: Transcriptional regulation is insufficient to explain substrate-induced flux changes in Bacillus subtilis
Source: Mol Syst Biol. 2013 Nov 26;9:709. doi: 10.1038/msb.2013.66 (PMC4039378; doi:10.1038/msb.2013.66)
Supplement: Supplementary Information — Supplementary Figures S1–5 [file msb201366-s1.pdf]

Supplementary Material for
"Transcriptional regulation is insufficient to explain substrate-induced flux changes in *Bacillus subtilis*"

Chubukov V., Uhr M, LeChat L, Kleijn R, Jules M, Link H, Aymerich S, Stelling J, Sauer U

Supplementary Figure S1 2
Supplementary Figure S2 3
Analysis of split ratios 4
Supplementary Figure S3 4
Supplementary Figure S4 5
Supplementary Figure S5 6
Supplementary Table 1 7
Supplementary Table 2 7
Supplementary Table 3 7
Supplementary Table 4 7
References 7

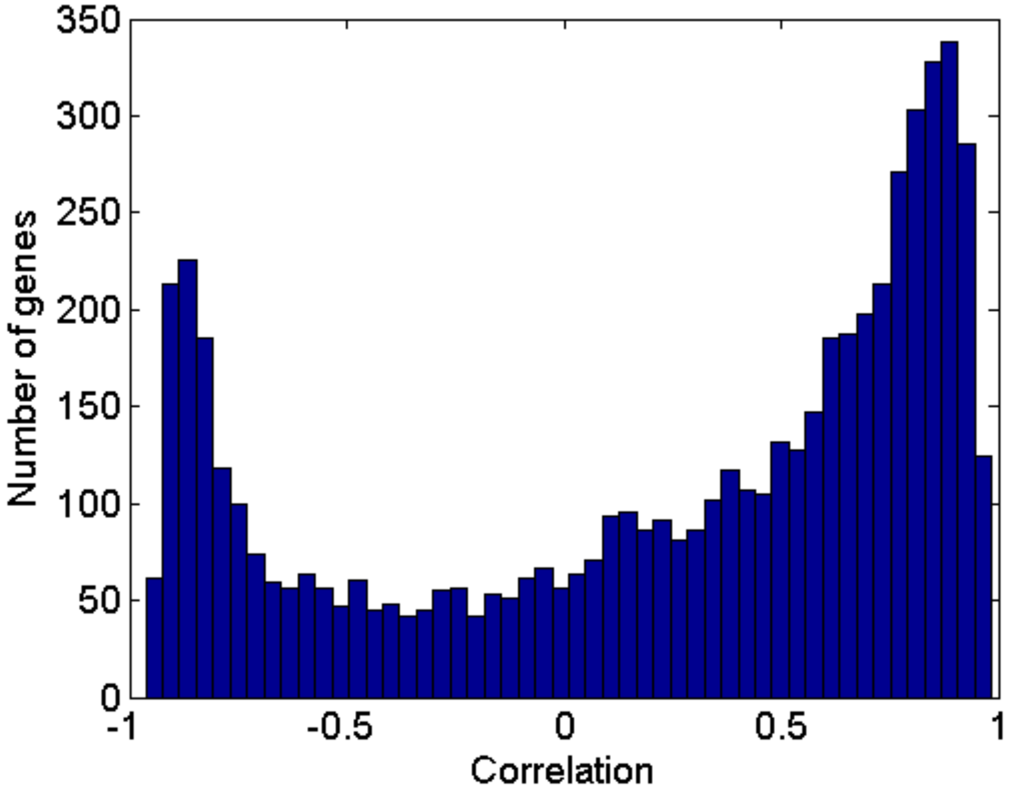

Supplementary Figure S1

Histogram of correlation between gene expression and growth rate. See Table S1 for enriched GO categories of growth-rate-correlated and anti-correlated genes.

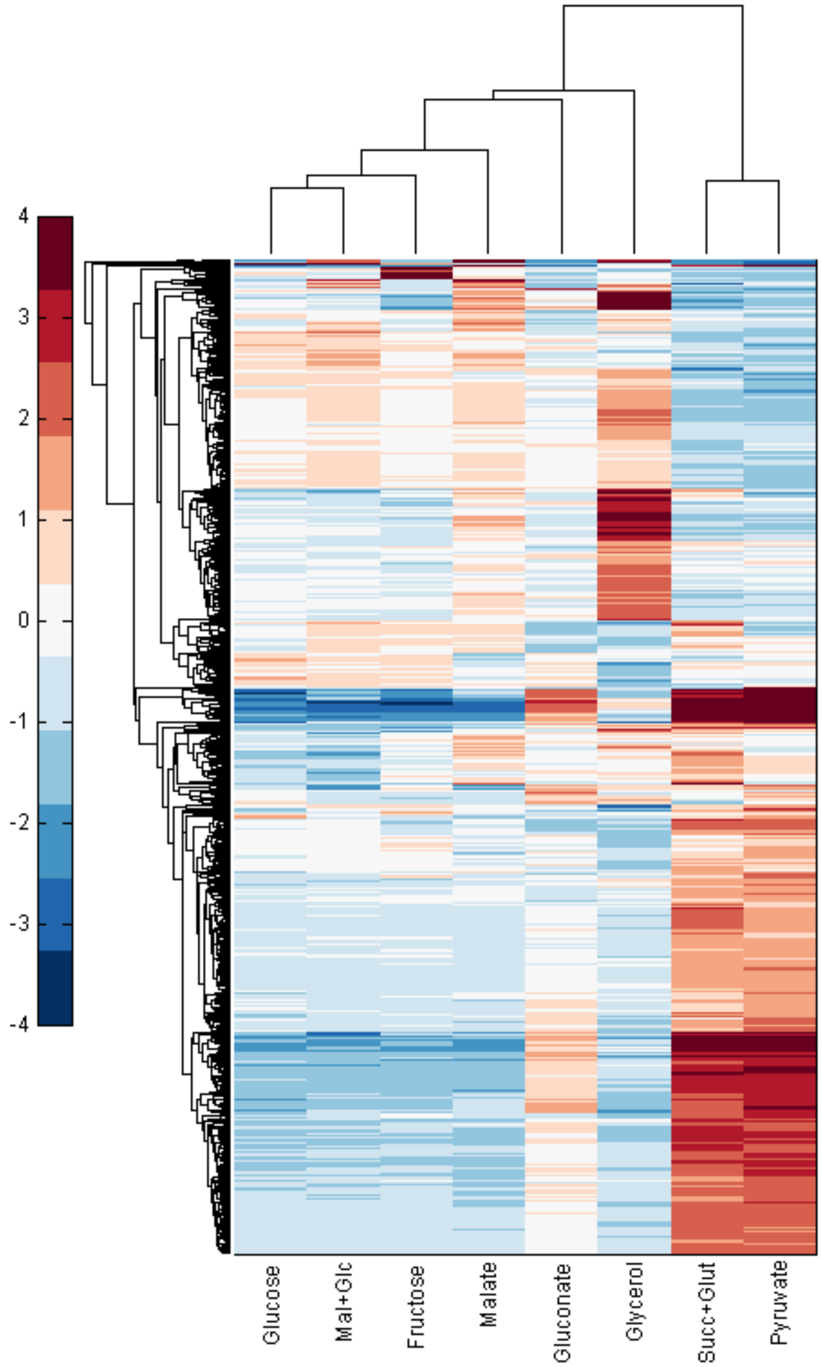

Supplementary Figure S2

Summary of gene expression changes for the 8 conditions. Genes with at least 4-fold difference between the minimum and maximum expression level are shown. Data are log_2-transformed and normalized to the mean for each gene.

Analysis of split ratios

We assume a flux J_0 that leads to the formation of metabolite S. S can then be converted by either of two irreversible enzymes, E_1 or E_2, and we call those fluxes J_1 and J_2 so that $J_0=J_1+J_2$. If both enzymes obey Michaelis-Menten kinetics

$$J_0 = E_1\, kcat_1 \left(\frac{S}{S + km_1}\right) + E_2\, kcat_2 \left(\frac{S}{S + km_2}\right) + \mu\, S$$

where μ represents dilution by growth (typically much smaller than flux and included only for mathematical consistency). We can solve for S analytically as a function of J_0, E_1, E_2, μ and the four kinetic parameters. We then obtain fluxes J_1 and J_2 as a function of those same parameters, using the solution for S.

We sample J_0, E_1, E_2, μ, km_1 and km_2 from log-normal distributions, and calculate the ratio $J_r= J_1/J_2$. To determine how this ratio changes with changes in enzyme concentration ratio, we perturb $E_r= E_1/E_2$. by a factor of 2 (typical enzyme changes seen in our data) while keeping other parameters constant, and calculate the ratio $\Delta J_r/ \Delta E_r$.

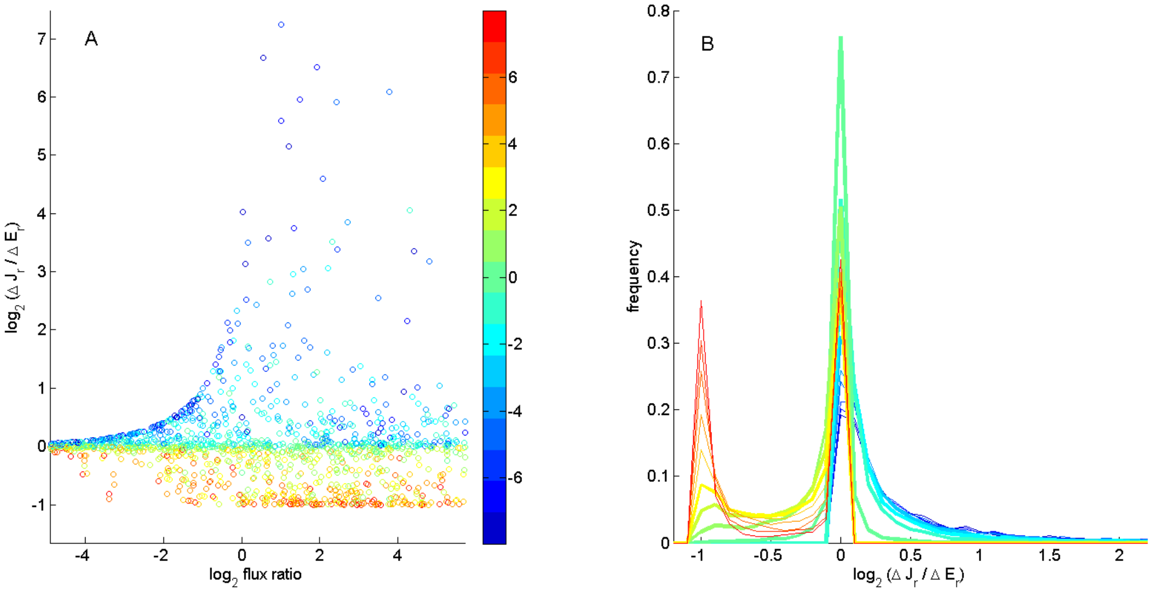

Supplementary Figure S3

Two ways of looking at the relationship between enzyme ratio and flux ratio. In Figure S3A, each point represents one simulated set of parameters. The x-value represents the original flux ratio J_r and the y-axis represents the difference between the fold change of the flux ratio and the fold change of the enzyme ratio. The color represents the $\log_2$ ratio km_1/km_2. In Figure S3B, histograms of the difference in flux ratio and enzyme ratio fold change are plotted for each bin of km_1/km_2 ratios (same colors as (A)). From (B), we conclude that for reasonably similar kinetic parameters, the flux ratio is approximately a linear function of the enzyme concentration ratio. Moreover, figure S3A shows that even in cases where the ratio km_1/km_2 is far from unity, if this is not compensated by enzyme abundance or turnover rate, so that J_1/J_2 is similarly far from unity in the opposite direction, flux ratio will still be approximately linear with enzyme ratio.

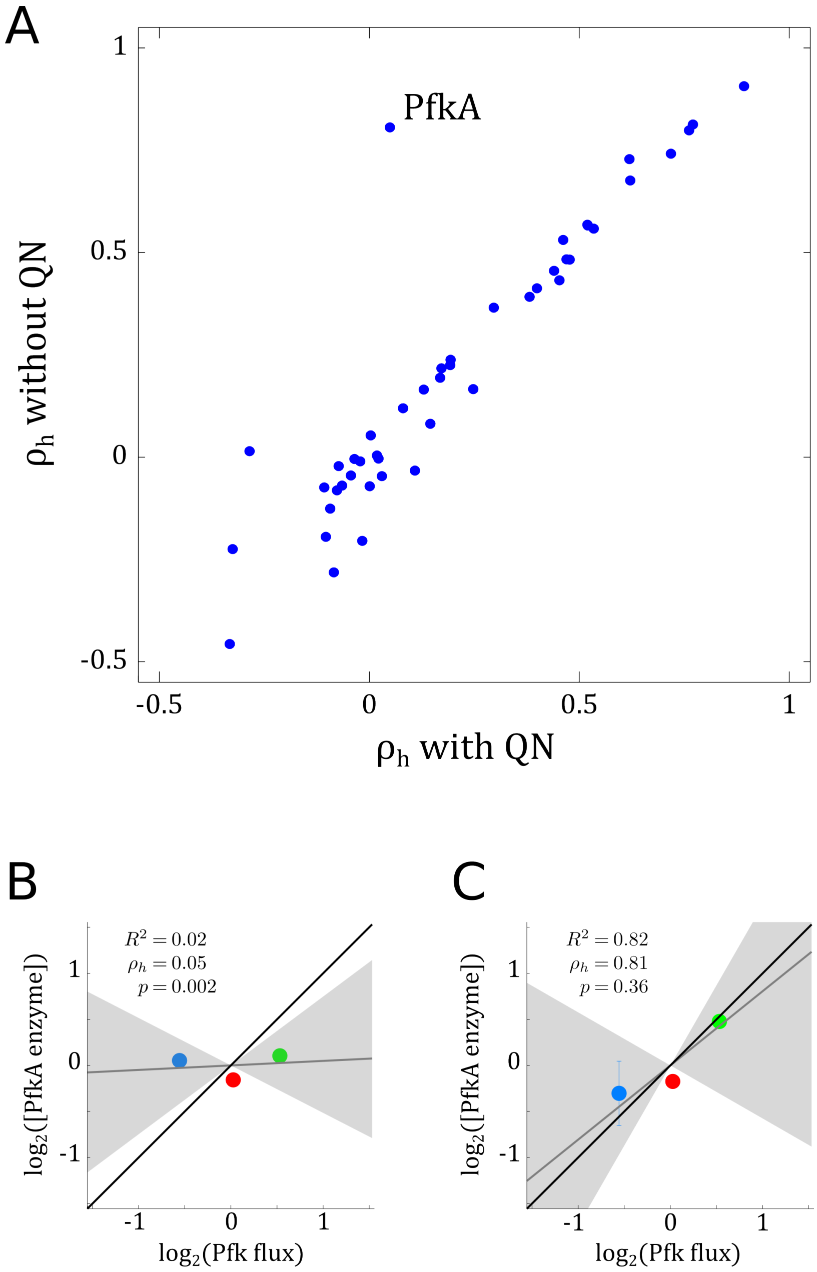

Supplementary Figure S4

Effect of quantile-quantile normalization (QN) on analysis of ρ_h. A) Identical analysis was performed with and without quantile-quantile normalization of gene expression data. Most effects were negligible – the only case in which the normalization caused a significant effect was for the calculated expression of PfkA during growth on gluconate, which resulted in a modified ρ_h as seen in (B) without QN and (C) with QN.

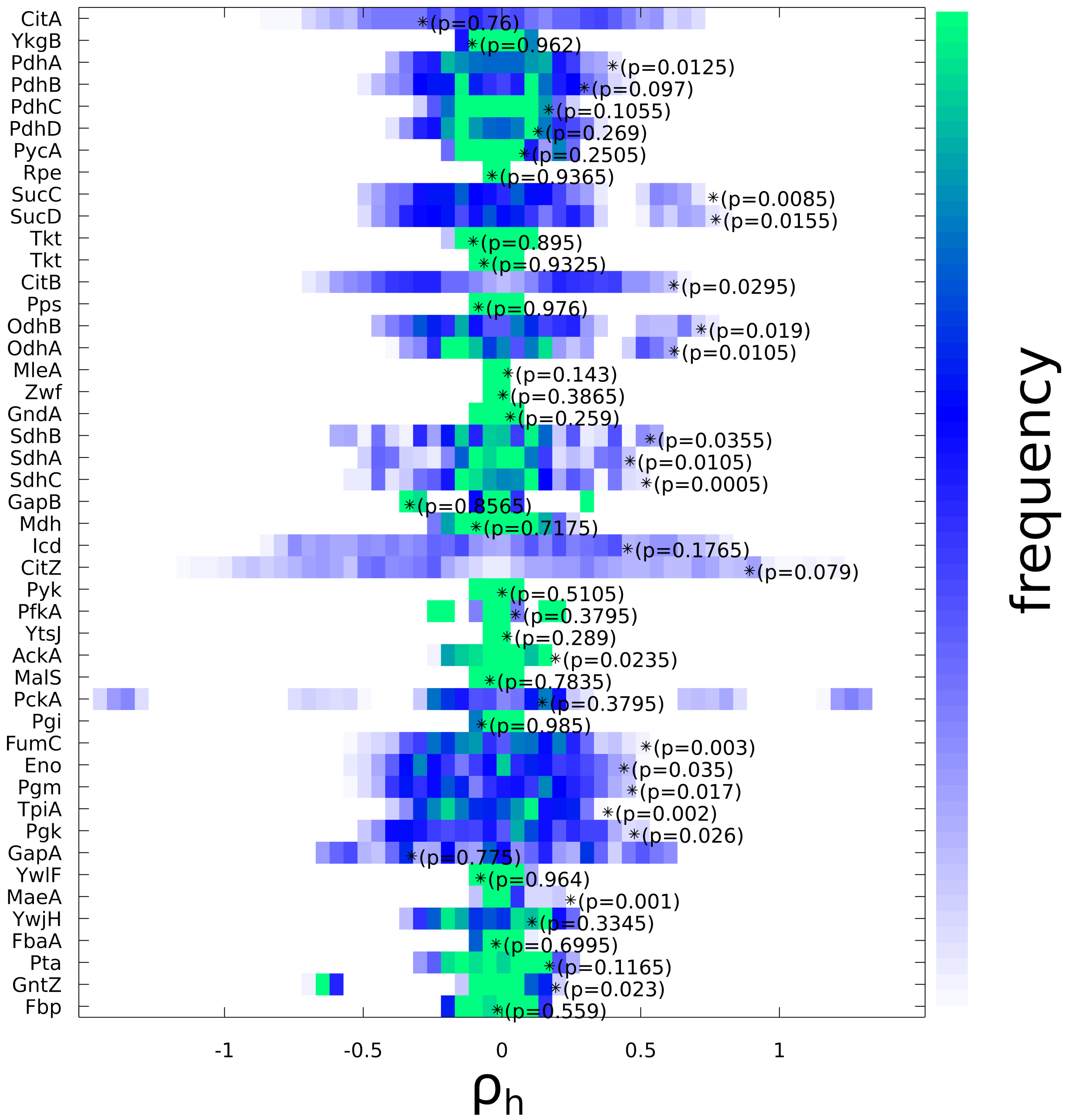

Supplementary Figure S5

Analysis of ρ_h under random permutations of expression data. For each enzyme, expression data was randomly assigned to conditions and ρ_h was recalculated. The histogram of resulting ρ_h values is shown as a colorbar in each row. The true ρ_h is shown as a black asterisk along with the empirical p-value. Even under randomly permuted expression values, many enzymes never achieve a high ρ_h simply due to the fact that enzyme expression changes were of a much lower magnitude than flux changes. However, for those enzymes where enzyme and flux changes had similar orders of magnitude, the true ρ_h tended to be far higher than expected by chance.

Supplementary Table 1

Gene ontology enrichment for growth-rate correlated (R>0.9) and anti-correlated (R<-0.9) genes. The genes in each list were analyzed for significant GO-term enrichment using gProfiler(Reimand et al, 2011). For each GO category, the following values are given: Q: number of genes in query (number of up-/down-regulated genes), T: number of genes in the GO category, Q&T: number of genes in both categories. Only statistically significantly enriched categories are shown.

Available for download as XLS file

Supplementary Table 2

Gene ontology enrichment for up- and down-regulated genes in each condition. A z-score was calculated for each gene and condition based on the mean and standard deviation across conditions. Genes with z >1 were called up-regulated and those with z <-1 were called down-regulated. The genes in each list were analyzed for significant GO-term enrichment using gProfiler(Reimand *et al*, 2011). For each GO category, the following values are given: Q: number of genes in query (number of up-/down-regulated genes), T: number of genes in the GO category, Q&T: number of genes in both categories. Only statistically significantly enriched categories are shown.

Available for download as XLS file

Supplementary Table 3

Metabolite concentrations in 8 conditions. All values are in mMol/L intracellular volume, calculated using a conversion factor of 1mL culture * 1 OD_{600} =0.89µL intracellular volume.

Available for download as XLS file

Supplementary Table 4

Central carbon fluxes in 8 conditions. All values are in mMol/gcdw/hr (gcdw=gram cell dry weight).

References

Reimand J, Arak T & Vilo J (2011) g:Profiler—a web server for functional interpretation of gene lists (2011 update). *Nucl. Acids Res.* **39:** W307–W315
